# Advancing ADMET prediction for major CYP450 isoforms: graph-based models, limitations, and future directions

**DOI:** 10.1186/s12938-025-01412-6

**Published:** 2025-07-23

**Authors:** Asmaa A. Abdelwahab, Mustafa A. Elattar, Sahar Ali Fawzi

**Affiliations:** Center for Informatics Science (CIS), School of Information Technology and Computer Science, Nile University, Juhayna Square, 26th of July Corridor, El Sheikh Zayed, Giza 12677 Egypt

**Keywords:** CYP enzymes, ADMET prediction, Drug discovery, Graph representation, Graph embeddings, Machine learning, Deep learning, Graph neural networks

## Abstract

Understanding Cytochrome P450 (CYP) enzyme-mediated metabolism is critical for accurate Absorption, Distribution, Metabolism, Excretion, and Toxicity (ADMET) predictions, which play a pivotal role in drug discovery. Traditional approaches, while foundational, often face challenges related to cost, scalability, and translatability. This review provides a comprehensive exploration of how graph-based computational techniques, including Graph Neural Networks (GNNs), Graph Convolutional Networks (GCNs) and Graph Attention Networks (GATs), have emerged as powerful tools for modeling complex CYP enzyme interactions and predicting ADMET properties with improved precision. Focusing on key CYP isoforms-CYP1A2, CYP2C9, CYP2C19, CYP2D6, and CYP3A4-we synthesize current research advancements and methodologies, emphasizing the integration of multi-task learning, attention mechanisms, and explainable AI (XAI) in enhancing the accuracy and interpretability of ADMET predictions. Furthermore, we address ongoing challenges, such as dataset variability and the generalization of models to novel chemical spaces. The review concludes by identifying future research opportunities, particularly in improving scalability, incorporating real-time experimental validation, and expanding focus on enzyme-specific interactions. These insights underscore the transformative potential of graph-based approaches in advancing drug development and optimizing safety evaluations.

## Introduction

CYP enzymes are a key class of proteins involved in the drug metabolism phase, which is itself a central component of absorption, distribution, metabolism, excretion, and toxicity (ADMET). Understanding the intricacies of drug metabolism, primarily facilitated by CYP enzymes, is crucial in the drug discovery life cycle. It helps in mitigating late-stage failures during the clinical phase, which often arise due to unfavorable ADMET properties [1–3]. These enzymes are pivotal in metabolizing a wide spectrum of compounds, making them a focal point in ADMET prediction models.

The current paradigm in ADMET research acknowledges that not all compounds are tested against all CYP isoforms due to the vast number of existing and newly synthesized chemical entities. High-throughput screening techniques using in vitro probe cocktails have been developed to simultaneously test multiple CYP isoforms. These cocktails are designed to determine the inhibition profiles of compounds, which are essential for predicting drug--drug interactions (DDIs) and potential adverse effects. Despite these advancements, the continuous synthesis of new compounds and the evolving understanding of CYP isoforms necessitate ongoing assessments [4–6].

Moreover, the development of selective probes and inhibitors for these isoforms has enhanced our ability to study them both in isolation and in combination. However, due to the chemical diversity of substrates and the potential for compounds to interact with multiple CYP isoforms, there are intrinsic limitations that complicate the creation of a comprehensive inhibition profile for every compound [7].

**Fig. 1:**
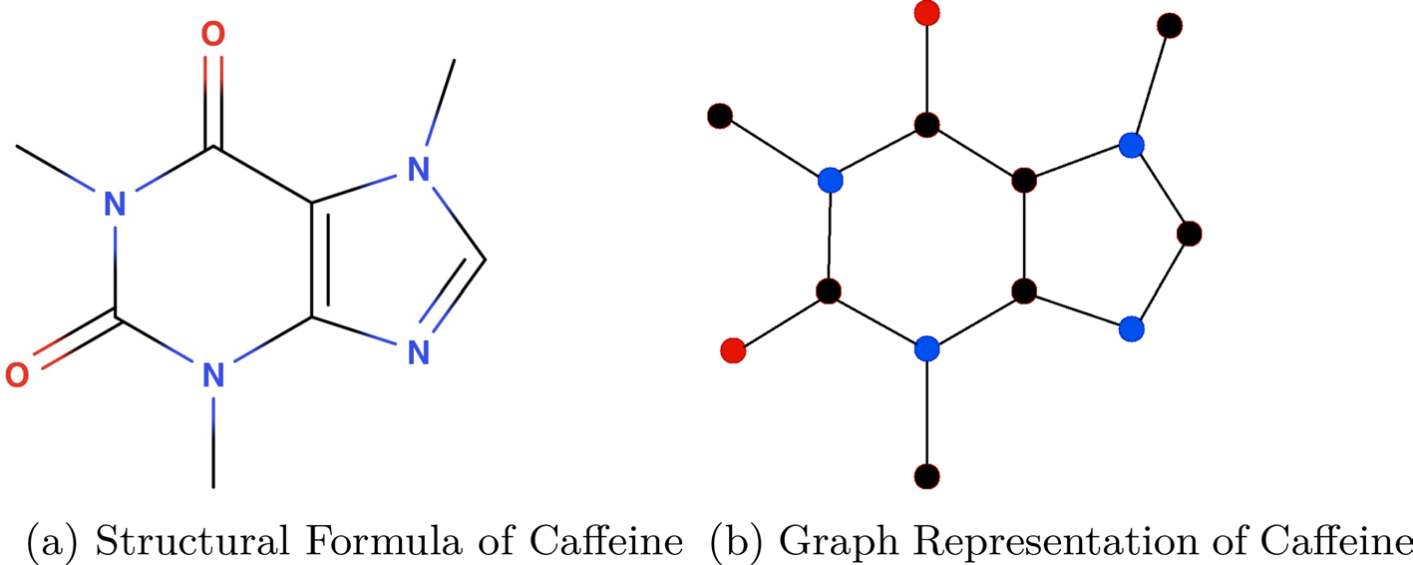
The diagram presents a streamlined representation of the caffeine molecule, where hydrogen atoms and bond types are omitted for clarity. This abstracted view facilitates a focus on the core atomic structure and connectivity within the molecule

In this context, computational modeling has emerged as a pivotal strategy in ADMET prediction involving CYP enzymes. With an influx of high-quality, voluminous data, machine learning (ML) and deep learning (DL) methodologies have revolutionized drug discovery and development [8, 9]. Graph-based modeling distinguishes itself within the array of computational methodologies due to its remarkable aptitude for representing molecular structures [10]. In this paradigm, molecules are elegantly depicted as graphs, where atoms are denoted as nodes interconnected by bonds (edges). This intuitive and organic representation aligns seamlessly with graph-based computational algorithms, facilitating the exploration and analysis of molecular configurations [11]. An example of this can be observed in Fig. 1, which demonstrates the caffeine molecule in a simplified graph format.

**Fig. 2:**
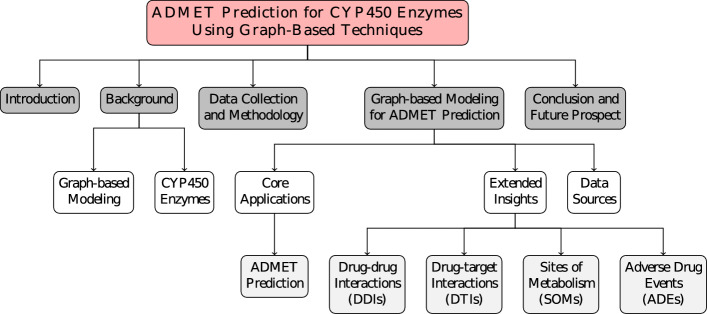
The figure outlines the key sections of the study, including an introduction to the topic, background on CYP enzymes and graph-based modeling, data collection and methodology, recent advancements in ADMET prediction using GNNs and XAI, insights from graph-based studies such as DDIs and drug--target interactions (DTIs), as well as data sources and future directions for research

The upcoming section will introduce graph modeling and detail the particular facets of the CYP enzymes that are pertinent to this review. This section sets the stage for the detailed discussion to follow. Additionally, Fig. 2 provides a graphical summary that encapsulates the essential themes and arguments to be detailed in this review, serving as a conceptual map for the content ahead.

## Background

### CYP enzymes

The significance of cytochrome P450 (CYP) enzymes in drug metabolism cannot be overstated, as more than 75% of clinically used drugs are metabolized through these pathways [12]. Drug metabolism occurs in two primary phases: Phase I, which is largely catalyzed by CYP enzymes, involves the chemical modification of drug molecules to increase their polarity and hydrophilicity. Phase II then involves conjugation reactions that further enhance solubility and facilitate excretion.

The human CYP enzyme system is encoded by 57 genes across 18 families, representing a complex and diverse set of metabolic pathways [12–15]. Among these, five isoforms---CYP1A2, CYP2C9, CYP2C19, CYP2D6, and CYP3A4---are particularly noteworthy, as they account for the majority of drug oxidation reactions in the liver. These enzymes transform lipophilic drugs into more water-soluble forms suitable for elimination [16, 17]. The activity of CYP enzymes varies significantly among individuals due to genetic polymorphisms, which can influence both drug efficacy and the risk of adverse reactions [18].

CYP enzymes interact with drugs in two distinct ways: as metabolic substrates, where the enzyme facilitates drug clearance, and as inhibitors (perpetrators), where the compound impedes the enzyme’s function and alters the metabolism of co-administered drugs. Predicting substrate potential is critical for dose optimization and understanding pharmacokinetics, whereas identifying inhibitors is essential for evaluating drug--drug interaction (DDI) risks. While many machine learning models address both endpoints, treating substrate and inhibitor predictions as separate tasks improves interpretability and aligns more closely with clinical and regulatory needs.

In this review, we focus on the five major CYP isoforms and examine how graph-based machine learning methods have been applied to predict their interactions with drug compounds. By evaluating the strengths and limitations of recent modeling approaches, we aim to highlight opportunities for more precise and interpretable ADMET prediction.

### Graph-based modeling

In the dynamic landscape of data science and computational modeling, graph-based modeling stands out as a transformative approach. Tracing its roots back to ancient diagrammatic practices and the concept of graphs in Ancient Greece, this technique has evolved into a pivotal tool in contemporary scientific research. It is characterized by representing entities as nodes and their interrelations as edges, enabling the illustration of intricate networks within diverse datasets in an accessible and visually compelling manner. Today, graph-based modeling is essential in various disciplines, from optimizing communication networks to deciphering biological complexities. Its broad applications underscore its versatility and critical role in addressing cross-disciplinary challenges, positioning it as a key player in both historical and modern contexts of knowledge expression and problem-solving.

Graph-based models have carved a niche in various domains, including social network analysis, bioinformatics, and recommendation systems. They excel in interpreting interconnected data, fostering informed decisions and complex problem-solving. By elucidating data structures and relationships, graphs facilitate profound insights and precise predictions [19]. Enhancing these models, graph analytics applies methods like centrality analysis and community detection [20], as well as motif analysis [21]. These techniques are instrumental in discerning patterns and influencers within large datasets, often uncovering insights unattainable through traditional analytical methods. Nonetheless, challenges such as scalability and intricate graph structures persist in graph analytics.

Graph-based modeling utilizes advanced graph algorithms, which are pivotal for solving complex problems in large networks, such as finding the shortest path or grouping similar items. These algorithms, however, face challenges in handling vast networks, prompting research into enhancing their efficiency [22]. Within the scope of ADMET prediction, these algorithms, including GCNs and GATs, effectively translate molecular graphs into detailed, low-dimensional embeddings. This transformation is promising for accurately modeling drug--CYP enzyme interactions and assessing ADMET properties of new compounds [23–25].

The forthcoming section will delve into a comprehensive review of how graph-based modeling has been employed in the field of ADMET prediction, particularly concerning the main five isoforms of CYP enzymes over the past Six years. This exploration will not only shed light on the contribution of graph-based methodologies to our understanding and prediction of drug metabolism and interactions, but also discusses the datasets used, the challenges and limitations encountered, and the prospective future directions in this field. This review aims to encapsulate the impact of these techniques in pharmacology and bioinformatics, illustrating both their current utility and potential for future advancements.

## Data collection and methodology

**Fig. 3:**
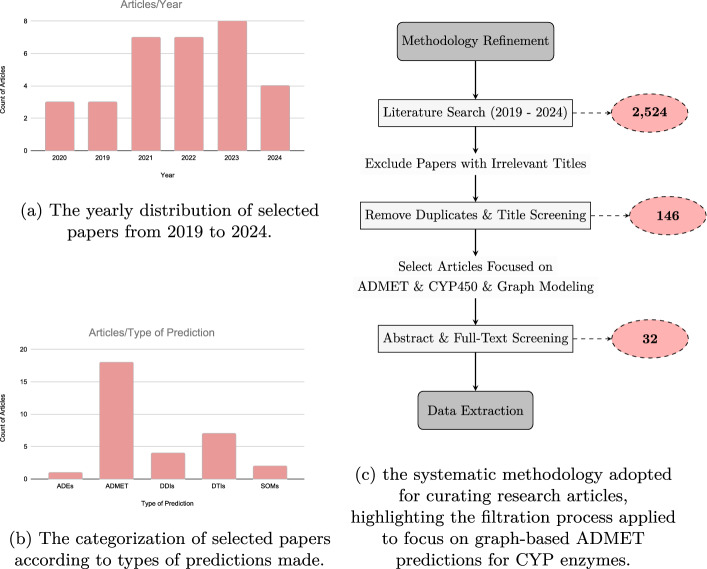
Data collection and article distribution analysis

Our literature review followed a structured approach to identify relevant studies from 2019 to 2024. The databases chosen for our search were Google Scholar, PubMed, and Scopus, selected for their specific strengths and coverage. Google Scholar provided a broad and extensive collection of scholarly articles, including grey literature and conference papers, offering a comprehensive view of the field. PubMed, a key database for biomedical literature, was crucial for its focused collection of life sciences and biomedical research, ensuring we captured relevant studies specifically related to ADMET and CYP enzymes. Scopus was included for its wide multidisciplinary coverage and excellent tools for citation analysis, aiding in the identification of influential and highly cited works in the domain of graph-based methods in drug discovery. The queries were designed to capture a broad spectrum of articles related to ADMET prediction for the major isoforms of CYP enzymes, utilizing graph-based modeling techniques. Specifically, the PubMed query incorporated a combination of key terms within the title and abstract fields that spanned descriptors of ADMET processes and ML terms, bounded by the publication dates from 2019 to 2024 and filtered to exclude reviews and meta-analyses. Similarly, targeted searches on Google Scholar and Scopus were conducted with analogous parameters. These searches were tailored to include only articles in English and were structured to ensure a comprehensive collection of relevant current literature. The search strings were constructed to balance specificity with breadth, allowing for the inclusion of articles that address the intersection of ML with ADMET Prediction and CYP enzymes. Searches across Google Scholar, PubMed, and Scopus yielded a combined total of 2,524 articles. Our two-stage screening involved title/abstract analysis for relevance, followed by full-text review for studies specifically using graph-based methods. For a detailed overview of the search and screening process, refer to Fig. 3.

While our search strategy aimed for comprehensive coverage, several limitations should be acknowledged. First, restricting searches to English-language publications may have excluded relevant studies published in other languages. Second, although Google Scholar, PubMed, and Scopus provide extensive and complementary coverage, there is a possibility that some pertinent studies indexed in other databases (e.g., Web of Science or IEEE Xplore) may have been missed. Third, our exclusion of review articles and meta-analyses, while intended to focus on original contributions, may have led to the omission of valuable synthesized insights. Finally, the manual screening process, despite being rigorous, may be subject to subjective interpretation during the inclusion/exclusion decision-making. These limitations could influence the completeness of the identified body of work and may introduce bias in the assessment of the current state of graph-based ADMET modeling for CYP enzymes.

## Recent advances in ADMET prediction

**Fig. 4:**
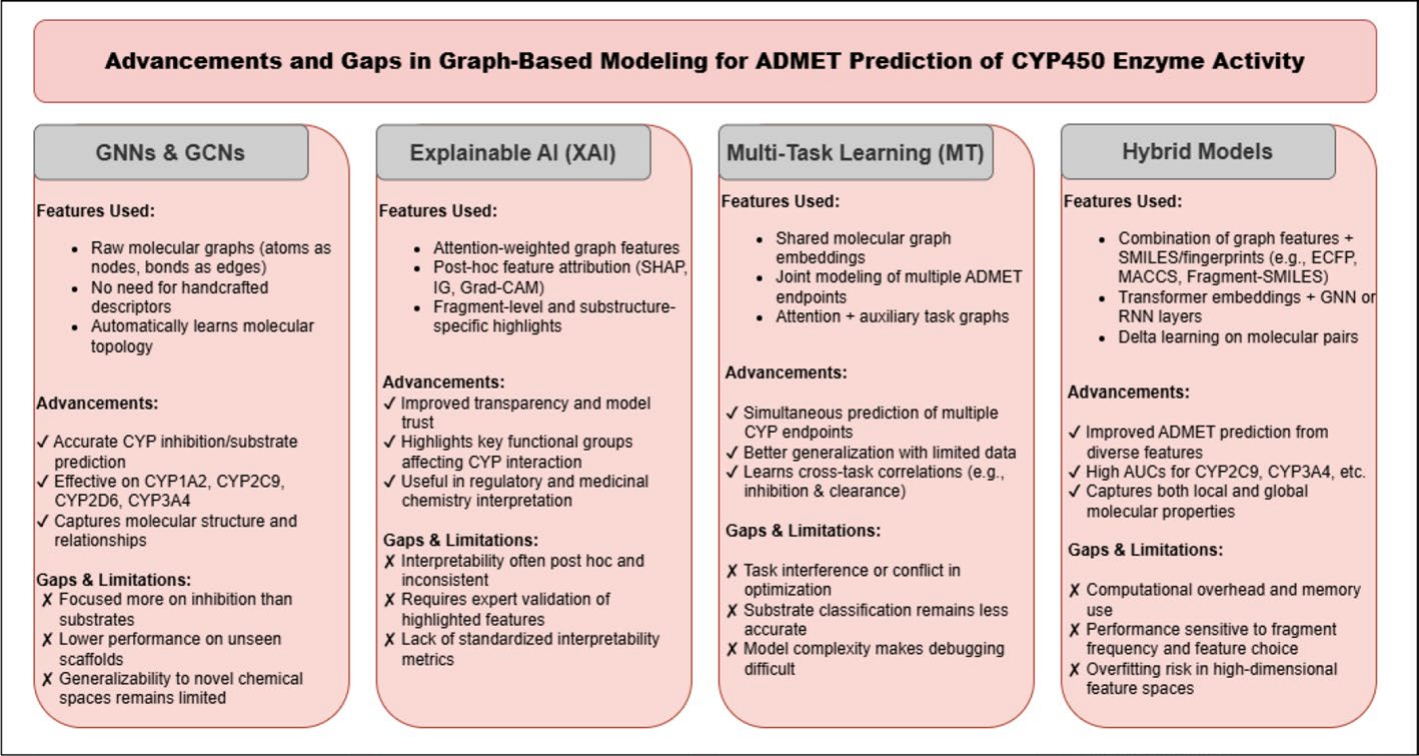
Summary of advancements and limitations across four major approaches in graph-based modeling for ADMET prediction of CYP450 enzyme activity: GNNs/GCNs, XAI, multi-task learning (MTL), and hybrid models. Each column outlines core features, recent advancements, and associated limitations for each modeling strategy

In recent years, significant advancements have been made in developing ML models to predict ADMET properties, with a strong focus on improving drug safety, efficacy, and metabolic profiles. This section categorizes these advancements into four key areas: GNNs and GCNs, XAI and interpretable graph-based models, multi-task learning with GNNs, and hybrid modeling approaches. Each category highlights how different modeling techniques are applied to predict ADMET properties, particularly in relation to CYP enzymes, which are central to drug metabolism and potential DDIs. For a detailed overview of the research papers on ADMET prediction, refer to Table 1 and Fig. 4.

Performance metrics reported in the table include AUROC (area under the receiver operating characteristic curve), AUPR (area under the precision--recall curve), F1-score, accuracy (ACC), balanced accuracy (BA), R^2^ (coefficient of determination), Matthews correlation coefficient (MCC), Pearson’s correlation coefficient (r), and sensitivity (Sn). These metrics are commonly used to evaluate classification and regression models in ADMET prediction tasks, particularly for CYP inhibition and substrate classification, reflecting both predictive performance and generalization ability.

### Graph neural networks and graph convolutional networks

GNNs and GCNs have emerged as powerful tools for predicting ADMET properties, particularly in the context of CYP enzyme interactions. These models treat molecular structures as graphs, where atoms serve as nodes and bonds as edges, enabling the automatic learning of molecular features from raw structural data without relying on traditional, handcrafted descriptors. One of the earliest examples of such an approach is Chemi-Net, which utilizes a GCN to improve the accuracy of ADMET property predictions by directly learning molecular features from graph-structured inputs. Despite its success in general ADMET tasks, Chemi-Net does not explicitly target CYP enzyme interactions. This suggests potential for further refinement in enzyme-specific applications, particularly in addressing challenges related to dataset noise and isoform resolution [26]. Building on the strengths of graph-based learning, the development of enterprise-wide predictive models, such as the gTPP model, further expanded the use of GCNNs for ADME predictions, particularly in inhibiting key CYP enzymes such as CYP1A2, CYP2D6, and CYP3A4. Trained on large compound datasets, the model achieved high predictive performance in external validation and surpassed several commercial tools in key metrics. However, as with many predictive models, challenges remain in extending performance to novel chemical scaffolds and improving the prediction of continuous metabolic endpoints such as clearance [27].

A significant advancement in this field is the introduction of attention mechanisms within GNN models, which improve interpretability and performance. A Unified GCNN model with an attention mechanism demonstrated high accuracy in predicting inhibitors for multiple CYP enzymes, including CYP3A4 and CYP2D6, by identifying critical molecular substructures responsible for CYP inhibition [28]. Similarly, the DeepCYPs platform employed a GNN-based architecture with attention mechanisms. Specifically, it utilizes a multi-task FP-GNN framework that combines graph neural networks with attention-enhanced fingerprint analysis to predict multiple CYP-related activities, outperforming traditional models like random forests (RFs) and support vector machines (SVMs). This approach allowed for high predictive accuracy across several major CYP isoforms, with the added benefit of enhanced interpretability through the identification of key molecular features [29].

The utility of GNNs in predicting CYP enzyme interactions has also been validated through comparative studies, such as the evaluation of ML models for CYP3A4, CYP2D6, and CYP2C9 inhibition. In this context, GNNs consistently outperformed traditional models, demonstrating the effectiveness of graph-based approaches in modeling complex enzyme--substrate relationships.

### Explainable AI and interpretable graph-based models

In recent years, there has been significant progress in integrating XAI techniques into graph-based models for ADMET property prediction, with a particular emphasis on improving the transparency and interpretability of ML algorithms. This is especially critical in drug discovery, where understanding the basis of model predictions supports both regulatory compliance and clinical translation.

Jiménez-Luna et al. [30] proposed a graph neural network model augmented with explainable AI, introducing a “molecular coloring” approach that highlights atom-level contributions to ADMET endpoints such as CYP3A4 inhibition. This method provides intuitive visual explanations using integrated gradients, improving model interpretability and aiding rational molecular design. Similarly, Rao et al. [31] combined GNNs with graph attention mechanisms to pinpoint influential substructures in toxicity and stability predictions, balancing interpretability with predictive performance. By incorporating these XAI-derived substructures as additional molecular fingerprints, their approach improved predictive performance across multiple benchmarks while enhancing model interpretability.

To further build trust in AI-driven predictions, Tariq [32] incorporated attribution techniques such as Integrated Gradients, Saliency maps, and GNNExplainer to identify important molecular substructures driving model decisions. This was particularly applied to explain predictions related to CYP2C9 inhibition, where catechol groups were identified as key contributors to inhibitory activity. Such post hoc interpretability efforts help contextualize GNN-based predictions, especially in critical applications like toxicity profiling and enzyme inhibition.

Beyond post hoc methods, Vangala et al. [33] proposed a novel approach that integrates domain-specific chemical knowledge directly into model design. By introducing a fine-grained fragmentation method (pBRICS) and constructing fragment-level molecular graphs, they enabled the development of interpretable multi-task GNN models for ADMET property prediction. The use of Grad-CAM allowed the identification of chemically meaningful substructures linked to predictive outcomes, while matched molecular pair (MMP) analysis validated the relevance of explanations. Their framework highlights the benefits of embedding chemical expertise into model representation to improve both accuracy and interpretability. In a multitask learning framework, Fang et al. [34] demonstrated how attention-based models can not only predict interactions with multiple CYP isoforms, but also reveal interpretable patterns underlying enzyme--substrate specificity by integrating attention mechanisms and SHAP.

### Multi-task learning with graph neural networks

In recent years, multi-task learning frameworks combined with GNNs have shown significant promise in predicting ADMET properties, particularly in relation to CYP enzyme interactions. These models leverage GNNs’ capacity to represent molecular structures as graphs and apply multi-task learning to predict multiple properties simultaneously. For instance, ADMETLab 2.0 utilizes a multi-task graph attention network to predict various ADMET endpoints, with a specific focus on CYP inhibition and substrate interaction, offering high predictive accuracy across multiple properties, especially for major enzymes like CYP3A4 and CYP2D6 [35]. HelixADMET similarly incorporates self-supervised pre-learning and multi-task fine-tuning frameworks, providing flexibility in predicting a wide range of ADMET properties, including metabolism-related endpoints. The model’s ability to use unlabelled data improves performance and extends to new prediction tasks [36]. Additionally, The MTGL‑ADMET framework (‘one primary, multiple auxiliaries’) enhances CYP inhibition modeling by employing an adaptive auxiliary-task selection strategy based on status theory and maximum-flow analysis. This mechanism dynamically identifies tasks with complementary signal while down-weighting less informative ones, yielding stronger predictive accuracy and reducing the influence of unrelated tasks [37]. This method strengthens the model’s generalization to various ADMET properties and proves particularly effective in predicting metabolic pathways. DeepCYPs, on the other hand, integrates molecular graph and fingerprint-based learning with an attention mechanism to improve prediction accuracy and interpretability for CYP inhibitory activity. It demonstrated state-of-the-art performance across five major CYP isoforms (CYP1A2, CYP2C9, CYP2C19, CYP2D6, and CYP3A4), achieving top AUC scores in four out of five cases. The attention-guided interpretability module highlights relevant substructures contributing to inhibition. While interpretability aids understanding, predictions are computational and benefit from experimental validation for further confidence [29].

### Hybrid modeling approaches combining graph-based and other molecular representations

In recent years, hybrid modeling approaches that combine graph-based methods with other molecular representations, such as molecular fingerprints or Simplified Molecular Input Line Entry System (SMILES) notation, have emerged as powerful tools for ADMET property prediction. These models leverage the strengths of different molecular representations to capture both local and global structural features, which enhances the predictive accuracy for complex tasks such as drug metabolism and toxicity. For instance, the study by Notwell [38] explored the use of multiple types of molecular fingerprints, including Extended Connectivity Fingerprints (ECFP), Avalon, and extended reduced graph approach (ErG)—with 200 molecular descriptors to enhance ADMET property prediction. Using CatBoost as the primary machine learning model, their framework achieved strong generalization across 22 benchmarks from the Therapeutics Data Commons, including metabolism-related endpoints involving CYP enzymes such as CYP3A4 and CYP2D6. The incorporation of GIN-based graph neural network fingerprints further improved performance in most tasks. While deep learning models like Chemprop and ChemBERTa were also evaluated, the fingerprint-based ensemble consistently outperformed them across CYP inhibition tasks. Notably, the model maintained a controlled feature dimensionality (2863 features) despite combining multiple representations, although overfitting in validation folds was acknowledged as a minor limitation [38]. Another study, DeepDelta (2023) [39], adopted a different hybrid approach by integrating GNNs with delta learning in a pairwise D-MPNN architecture to predict how small structural modifications in molecular derivatives would affect ADMET properties. This model demonstrated high predictive accuracy, particularly in predicting the impact of structural changes on interactions with CYP enzymes, making it highly effective in guiding drug optimization efforts [39]. Similarly, Aksamit [40] proposed a novel hybrid representation strategy—Hybrid Fragment-SMILES Tokenization (HFST)—which combines high-frequency molecular fragments with character-level SMILES encoding. This hybrid input was used to train a Transformer-based multi-task model (MTL-BERT) for ADMET prediction. Without using graph neural networks (GNNs), their approach still achieved competitive or superior performance on metabolism-related tasks, including CYP3A4 and CYP2D6 inhibition and substrate classification. Notably, when benchmarked against deep learning models like Chemprop and AttentiveFP—both of which use GNNs—the HFST model demonstrated stronger generalization in several tasks, highlighting the strength of hybrid tokenization even in the absence of explicit graph-based input [40]. These hybrid approaches not only improve predictive performance, but also offer insights into drug metabolism and toxicity, facilitating more informed decision-making in drug discovery.

Despite methodological diversity across graph-based models-ranging from GNNs and GCNs to hybrid and explainable architectures, several recurring challenges are evident. A common limitation is the constrained generalizability of models to novel chemical entities, often due to reliance on public datasets with limited chemical diversity. Additionally, many models face scalability issues and computational overhead, especially when integrating multiple molecular representations or explainability components. Interpretability remains another cross-cutting concern; although attention mechanisms and XAI techniques offer promise, their standardization and validation in regulatory contexts are still evolving. These shared limitations underscore the need for benchmark datasets, model transparency, and integrated evaluation pipelines that reflect real-world variability in drug metabolism.

**Table 1:** Comparison of studies on CYP enzyme prediction and ADMET properties

Study	CYP isoforms studied	Datasets/Data sources	Methodology	Key findings & Notes
[26]	CYP3A4 (part of a broader study covering five ADME endpoints)	**Tox21**: Toxicology in the 21 st Century high-throughput screening program; **PubChem BioAssays**: A subset of the PubChem database maintained by the NIH.	Deep learning model combining molecular graph convolutional networks with multi-task deep neural networks (MT-DNN) for ADME property prediction.	**Key findings:** Chemi-Net outperformed Cubist in both CYP450 inhibition datasets. R^2^ improved from 0.597 to 0.692 (small, clean dataset) and from 0.315 to 0.414 (large, noisier dataset), showing enhanced predictive accuracy for CYP450 inhibition (likely CYP3A4). **Notes:** While CYP3A4-related data was included, exact isoform-specific modeling was not the study’s primary focus. Additionally, MT-DNN was not directly applied to the CYP450-specific inhibition subsets, which may influence results depending on dataset noise and heterogeneity.
[35]	CYP1A2, CYP3A4, CYP2C9, CYP2C19, CYP2D6.	Chemical Database of Bioactive Molecules (ChEMBL), PubChem, OCEHM, peer-reviewed literature, and freely accessible software Toxicity Estimation Software Tools (TEST) developed by the U.S. Environmental Protection Agency [41, 42].	Multi-task graph attention network to predict ADMET endpoints.	**Key findings:** ADMETlab 2.0 integrates 88 ADMET-related endpoints using multi-task graph attention (MGA) models. For CYP450 metabolism classification models, inhibitors achieved high AUCs (e.g., CYP1A2: 0.928, CYP2C9: 0.919, CYP3A4: 0.921), while substrate predictions were less accurate (e.g., CYP1A2: 0.737, CYP2C9: 0.725, CYP3A4: 0.776). On average, classification models reached AUC = 0.863 and MCC = 0.53, with 27 models having ACC > 0.8. Regression models achieved average R^2^ = 0.783. **Notes:** Some models (e.g., LC50DM and clearance) had limited data and underperformed. Leave-cluster-out validation confirmed generalizability with avg. MCC = 0.47 (classification) and R^2^ = 0.65 (regression). Models were faster and more scalable than previous versions but substrate prediction remains less robust than inhibitor prediction for CYP endpoints.
[27]	CYP1A2, CYP2C8, CYP2C9, CYP2C19, CYP2D6, CYP3A4.	**Proprietary:** in-house compound screening data; **Public:** ChEMBL + DrugBank	GCNN with multitask learning to predict CYP inhibition.	**Key findings:** gTPP model built using the Chemprop graph convolutional neural network and trained on up to 40,000 compounds per CYP isoform predicts 18 ADME endpoints including CYP1A2, 2C8, 2C9, 2C19, 2D6, and 3A4. External validation yielded: CYP3A4 inhibition - Validity = 0.92, Efficiency = 0.48 at 95% confidence ($\varepsilon = 0.05$). gTPP predictions showed higher ROC AUC and accuracy than commercial models like StarDrop and ACD/Labs - for example, CYP2C9 and 2D6 accuracy improved by +53% and +74%, respectively. Overall average across endpoints: 90% validity, 55% efficiency. Validity = fraction of confident predictions (confidence ≥ 95%); Efficiency = accuracy among those confident predictions. **Notes:** The model used binary inhibition classification and did not account for substrate-specific dynamics or $\textrm{IC}_{50}$ variability. Generalization to novel chemical scaffolds remains an area for improvement.
[30]	CYP3A4.	PubChem BioAssays including inhibitors and substrates (AID: 1851, 1024, 1025)	Integrated gradients-based XAI with GNNs for predicting ADMET properties	**Key findings:** XAI-based GNN model trained to classify drug-like molecules by their CYP3A4 inhibition potential. Using integrated gradients as an attention-based substructure attribution method, the model provided interpretable "molecular coloring" visualizations that highlighted atom-level contributions. The model achieved AUROC = 0.85, comparable to standard GNN baselines. Attention-based substructure attribution aligned with known reactive moieties. **Notes:**While the approach achieved promising results, the authors noted moderate overall performance, the absence of external validation, and the need for caution in interpreting attention weights without supporting experimental evidence.
[31]	CYP3A4.	ChEMBL, Tox21, QM9.	GNNs with explainability techniques for molecular property prediction.	**Key findings:** The Communicative Message Passing Neural Network (CMPNN) achieved the highest AUROC (0.979) on the CYP450 benchmark, followed closely by GraphNET (0.976), with GAT (0.865) and GraphSAGE (0.864) performing moderately. Traditional ML baselines like XGBoost (0.868) and Random Forest (0.818) scored lower. The CMPNN combined with Integrated Gradients (IG) yielded the best interpretability, with high attribution accuracy in identifying toxic substructures, outperforming most medicinal chemists. **Notes:** The use of CYP450-related substructures derived from activity cliffs offers a promising route for interpretable modeling, though their applicability to more complex or noisy real-world datasets may vary. Attribution-based evaluations showed stronger performance on synthetic benchmarks (e.g., AUROC = 0.827) than on realistic data (e.g., AUROC = 0.566), suggesting potential sensitivity to dataset composition. As the approach relies on post hoc XAI methods and annotated datasets, future work could explore integrating experimental insights to further validate substructure relevance.
[28]	CYP1A2, CYP2C9, CYP2C19, CYP2D6, CYP3A4.	**PubChem (21 AIDs)** — Curated datasets from PubChem BioAssay with paired labeling for CYP1A2, 2C9, 2C19, 2D6, and 3A4 isoforms.	Hybrid model combining GCN (graph input) and 1D-CNN (SMILES input) with attention mechanism to predict inhibitors of five CYP450 isoforms.	**،Key findings:** The GCNN model combining GCN with attention and 1D-CNN achieved strong predictive performance on CYP1A2, 2C9, 2C19, 2D6, and 3A4 inhibitors, outperforming the iCYP-MFE model on 4 of 5 isoforms. AUROC ranged from 0.89 to 0.92 across isoforms with accuracy between 0.80–0.88. Attention-based interpretability enabled identification of key inhibitory substructures.**Notes:** the model showed lower sensitivity compared to specificity on imbalanced test sets and may be affected by variations in experimental annotations across datasets. Further refinement of binding site encoding and data quality may enhance generalizability.
[32]	CYP2C9.	DrugBank.	GNNs with explainability using Integrated Gradients and GNNExplainer.	**Key findings:** Among the CYP-related endpoints, the CYP2C9 substrate model failed due to severe data imbalance (8994 non-substrates vs. only 3 substrates) and was excluded from further analysis. For CYP2C9 inhibitors, classification models using MACCS fingerprints achieved strong performance with AUC = 0.9219 and MCC = 0.8716. GNN models for CYP2C9 inhibitors also performed well (average ACC = 0.93) and were explainable via Integrated Gradients and GNNExplainer, which identified catechol groups as key toxic substructures. **Notes:** Applicability Domain (AD) analysis revealed that 644 compounds were outside the AD for MACCS models, with test set coverage of only 34%, indicating limited generalizability for structurally novel CYP-related compounds. When out-of-domain data were used for testing, model AUC dropped from 0.92 to 0.80 and MCC from 0.86 to 0.65, confirming reduced reliability outside the AD.
[34]	CYP3A4, CYP2C9, CYP2C19, CYP2D6, CYP1A2.	Substrate datasets curated for five major CYP isoforms.	Multitask GNN and fingerprint-based models incorporating attention mechanisms and SHAP values for interpretability.	**Key findings:** The multitask model achieved strong performance (AUC = 90.8%) and demonstrated robustness even on relatively small datasets, such as those for CYP1A2, 2C9, and 2C19. The use of SHAP and attention provided interpretable insights, highlighting substructures aligned with known metabolic features, as supported by literature and substructure mining tools. **Notes:** Additionally, we kindly ask that the last two entries in the table be removed for clarity and relevance.
[36]	CYP1A2, CYP2C19, CYP3A4, CYP2D6, CYP2C9 (general metabolism).	Includes labelled and unlabelled data from ChEMBL, PubChem, DrugBank, and peer-reviewed scientific papers.	GNNs with self-supervised and multi-task learning to predict ADMET properties.	**Key findings:** H-ADMET demonstrated strong predictive performance across CYP450 enzymes, with AUCs for inhibitor/substrate classification as follows: CYP1A2 (0.948/0.949), CYP2C19 (0.939/0.945), CYP2C9 (0.934/0.944), CYP2D6 (0.905/0.956), and CYP3A4 (0.930/0.967). It outperformed admetSAR 2.0 and ADMETlab 2.0 on all overlapping CYP endpoints. The system employs a three-stage training framework that combines self-supervised and multi-task learning, enhancing generalization to novel molecular scaffolds. **Notes:** Substrate classification tasks are relatively more complex than inhibition tasks due to limited training data and mechanistic ambiguity at the endpoint level.
[37]	CYP3A4, CYP2D6, CYP2C9.	Integrated dataset of 43,291 drug-like compounds across 24 ADMET-related endpoints (18 classification, 6 regression) derived from 8 publications. Endpoints span Absorption (5), Distribution (2), Metabolism (5), Excretion (2), Toxicity (8), and Physicochemical properties (2).	Multi-task graph learning with adaptive auxiliary task selection.	**Key findings:** MTGL-ADMET model demonstrated superior predictive performance for CYP inhibitors, outperforming state-of-the-art models across all tasks. For CYP1A2, CYP2C19, CYP2C9, CYP2D6, and CYP3A4 inhibitor prediction, AUC scores were 0.952, 0.804, 0.794, 0.869, and 0.916, respectively---higher than baseline single- and multi-task GNN models. The model adaptively selected auxiliary tasks using status theory and maximum flow, achieving better task-specific synergy. **Notes:** Attention-based interpretability provides valuable insights, though further refinement could help capture subtler molecular interactions—like nuanced hydrophilic/lipophilic influences. Also, enhancing clarity around the internal task-selection criteria could improve usability.
[29]	CYP1A2, CYP2C9, CYP2C19, CYP2D6, CYP3A4.	PubChem BioAssay (AID 1851, 410, 883, 889, 891, 884)	Multi-task FP-GNN model combining molecular graphs and fingerprint features	**Key findings:** Multi-task FP-GNN achieved state-of-the-art performance across five CYP isoforms (CYP1A2, CYP2C9, CYP2C19, CYP2D6, CYP3A4) with average AUC=0.905, F1=0.779, BA=0.819, MCC=0.647; ranked 1 st in AUC for 4/5 isoforms. **Notes:** While the model demonstrated robust generalizability, a performance drop was observed for compounds outside its applicability domain (e.g., AUC$\downarrow$ to 0.852, MCC$\downarrow$ to 0.510). The interpretability offered through attention mapping highlights key substructures, though predictions remain probabilistic and benefit from experimental confirmation.
[33]	CYP1A2, CYP3A4, CYP2D6, CYP2C9, CYP2C19.	ChEMBL, PubChem, DrugBank.	GNNs with domain-specific knowledge for improved explainability and accuracy.	**Key findings:** MT-FraRGCN model achieved strong performance: AUROC scores were 95.44 (CYP1A2), 81.11 (CYP2C19), 84.24 (CYP2C9), 85.41 (CYP2D6), and 88.45 (CYP3A4). These results slightly outperformed or matched ADMETLab2.0 across most CYPs. The fragment-based representation enabled interpretable predictions using Grad-CAM. **Notes:** While the model demonstrated modest performance gains over existing approaches, prediction outcomes were sometimes influenced by scaffold frequency. Additionally, matched molecular pair (MMP) validation was limited for CYP endpoints due to insufficient paired data.
[38]	CYP3A4, CYP2D6, CYP2C9.	ChEMBL, PubChem, Therapeutics Data Commons (TDC).	ML using gradient-boosted trees (CatBoost) with combined molecular fingerprints (ECFP, Avalon, ErG) and GNN-derived features (GIN) for ADMET prediction.	**Key findings:** The study evaluated model performance on 22 ADMET benchmarks from the Therapeutics Data Commons, including CYP enzyme inhibition tasks. The final model-CatBoost using a concatenation of ECFP (1024), Avalon (1024), ErG (315), 200 molecular descriptors, and GIN (300)-achieved strong generalization. Without GNN features, the model reached top-1 in 6/22 and top-3 in 16/22 benchmarks. After adding the GIN fingerprint, top-1 performance increased to 11/22 and top-3 to 19/22. These included benchmarks on CYP1A2, CYP2C9, CYP2C19, CYP2D6, and CYP3A4 inhibition. Compared to deep learning models (Chemprop, ChemBERTa, Grover), the fingerprint-based CatBoost model consistently outperformed across most CYP tasks. **Notes:** While extensive hyperparameter tuning was performed using Optuna, minor overfitting was observed in validation folds. Neural network-based fingerprints underperformed compared to traditional representations. Despite high dimensionality (~2863 features), the model maintained good generalization.
[39]	Microsomal and hepatic clearance endpoints affected by CYP450 activity.	ChEMBL, TDC.	GNNs with delta learning for predicting ADMET improvements in molecular derivatives.	**Key findings:** DeepDelta demonstrated improved performance on CYP-related ADMET tasks such as microsomal clearance (Pearson’s r: 0.468 vs. 0.451 ChemProp and 0.444 Random Forest). However, for hepatic clearance, it was the only task where DeepDelta was significantly outperformed (Pearson’s r: 0.392 vs. 0.431–0.438). This reflects challenges in modeling complex elimination pathways from limited data. **Notes:** DeepDelta excelled in predicting large property differences and scaffold-hopping cases but showed limitations on small changes and clearance endpoints, highlighting the need for more data to improve predictions on complex CYP-related properties.
[40]	CYP3A4, CYP2D6, CYP2C9, CYP2C19, CYP1A2.	ChEMBL, MOSES, ZINC-250K, TDC.	Hybrid Fragment-SMILES Tokenization with Transformer-based MTL-BERT model for ADMET prediction.	**Results:** The study employed the MTL-BERT Transformer model with a novel Hybrid Fragment-SMILES Tokenization (HFST) strategy to predict 29 ADMET endpoints, including several CYP inhibition and substrate classification tasks. Specifically, CYP inhibition prediction was conducted for CYP1A2, CYP2C9, CYP2C19, CYP2D6, and CYP3A4, while substrate classification tasks included CYP2C9, CYP2D6, and CYP3A4. Using a fragment frequency cutoff of 1000, HFST consistently outperformed standard SMILES tokenization across most CYP inhibition tasks. For example, AUROC scores for CYP2C19, CYP2C9, and CYP3A4 inhibition reached 0.96 each with HFST (one-phase). Substrate prediction also improved, with CYP2D6 and CYP3A4 substrate tasks achieving AUROCs of 0.899 and 0.84, respectively---both surpassing the SMILES baseline. **Limitations:** model performance declined when lower-frequency fragments were included, indicating sensitivity to fragment vocabulary. While HFST improved generalization and performance, limitations remain for complex or data-scarce endpoints such as hepatic clearance, underscoring the need for further tuning of token frequency thresholds and pretraining strategies.
[43]	CYP3A4, CYP2D6, CYP2C9, CYP2C19, CYP1A2.	ChEMBL, PubChem, DrugBank.	SVM models trained on molecular fingerprints (Morgan, MACCS, RDKit) for CYP450 inhibition prediction.	**Results:** The study developed SVM models to predict inhibition of five major CYP isozymes (1A2, 2C9, 2C19, 2D6, 3A4) using molecular fingerprints (Morgan, MACCS, RDKit). On independent test sets, Morgan fingerprints yielded the best performance, with balanced accuracy (BA) ranging from 0.81 to 0.85, Matthews correlation coefficient (MCC) between 0.61 and 0.70, and sensitivity (Sn) from 0.72 to 0.83. MACCS-Morgan combined models had slightly lower but comparable results (BA: 0.79–0.85, MCC: 0.59–0.69, Sn: 0.69–0.82). Compared to existing models (e.g., Plonka et al., 2021), the proposed SVM models showed improved or competitive performance, particularly for CYP1A2, 2C19, and 3A4, but slightly lower MCC for CYP2C9 and 2D6. **Limitations:** include reliance on imbalanced data and absence of ADME database data, which constrained overall performance despite competitive metrics.
[44]	CYP3A4, CYP2D6, CYP2C9.	ChEMBL, PubChem, DrugBank.	ML models (GNNs, RFs, SVMs) for CYP inhibition prediction.	**Results:** Systematic benchmarking on CYP3A4, CYP2D6, and CYP2C9 showed that XGBoost and CatBoost models using combined fingerprints and physicochemical descriptors achieved the best AUC (0.92); deep learning models were slightly inferior with average AUC of 0.89. **Limitations:** Evaluated only on binary inhibition; DL models underperformed classical ML; data volume and sampling strategy had minimal impact, but broader generalizability was not assessed.

## Extended insights from graph-based studies relevant to ADMET predictions

This subsection will highlight graph-based studies that, while not directly predicting ADMET properties, focus on related chemical properties and interactions---such as DDIs, DTIs, sites of metabolism, and adverse drug events (ADEs)---that have a significant impact on ADMET outcomes. These studies provide crucial insights into how chemicals behave in biological systems, indirectly influencing key ADMET characteristics like metabolism, distribution, and toxicity.

### Drug--drug interactions

Graph-based approaches have been widely used to predict DDIs, which can indirectly affect ADMET characteristics such as metabolism, distribution, and toxicity. These studies demonstrate how molecular structure-based representations can be leveraged to infer potential interactions between drugs.

Ryu et al. (2019) introduced a hybrid model combining artificial neural networks (ANNs) and graph similarity measures to predict DDIs. Drugs were represented as graphs, allowing the model to capture molecular-level relationships. This approach improved DDI prediction accuracy but did not include metabolic interaction mechanisms such as those mediated by CYP enzymes [45].

Zitnik et al. (2019) proposed graph-augmented convolutional networks (GACNs), integrating GCNs with attentive pooling to extract informative drug substructures. The model offered improved interpretability and predictive performance for DDIs [46].

Xu et al. (2021) developed attention-gated graph convolutional networks (AG-GCNs) to extract DDI information from structured FDA drug labels. Their architecture used attention mechanisms to identify the most relevant components, enhancing interpretability and DDI detection from textual data [47].

Zhang et al. (2022) introduced a link-aware graph attention network (LAGAT) that incorporated topological and semantic features to capture link-level drug relationships. This model achieved high DDI prediction accuracy using both node and edge features [48]. A summary of these key graph-based studies on DDI prediction—including their methodologies, datasets, and findings—is presented in Table 2.

**Table 2:** Summary of graph-based studies on DDIs

Study	CYP isoforms studied	Datasets/Data sources	Methodology	Key findings and Notes
[45]	None; general DDIs	DrugBank	A hybrid model that combines ANNs with classic graph similarity measures to predict DDIs. Drugs are modeled as graphs where nodes represent atoms and edges represent bonds, and graph similarity metrics are used to quantify molecular similarity. The ANNs then learn drug interactions using these graph-based features.	**Key findings:** The study evaluated AMF and AMFP models using DrugBank versions and removed interactions involving CYP enzymes (1A2, 2B6, 2C8, 2C9, 2C19, 2D6, 2E1, 3A4, 3A5, 3A7) to test generalizability beyond shared metabolism. Removing 56,874 CYP-related interactions (37.7% of total) reduced AUROC for AMFP from 0.807 to 0.775, and for AMF from 0.748 to 0.705, indicating some reliance on CYP-mediated interactions. The models outperformed the Vilar method (drop of 0.044 AUROC) and maintained relatively strong performance, suggesting they also capture pharmacodynamic effects, not only CYP-related metabolic interactions. **Notes:** Reduced performance when CYP-related interactions were excluded, indicating partial reliance on metabolic mechanisms but preserving reasonable generalizability.
[46]	CYP2D6; general DDIs	DrugBank	A graph-augmented convolutional network (GACN) model was proposed to enhance DDI prediction. The model integrates GCNs with attentive pooling to extract meaningful substructures from drugs. Drugs are represented as graphs, where the structure is learned using GCN, and attention mechanisms focus on key features for improved interpretability.	**Key findings:** The study proposed a GCNN-based model with an attentive pooling mechanism for predicting drug-drug interactions (DDIs), achieving strong overall performance (ROC: 0.988, F1-score: 0.956, AUPR: 0.986). A case study involving Celecoxib and Mephenytoin demonstrated the model’s ability to predict CYP2D6-mediated interactions with high confidence (prediction score: 0.97), supported by interpretability through attention-weighted atom highlighting. **Notes:** The model was evaluated primarily on a general DDI dataset and not specifically benchmarked across individual CYP isoforms, limiting direct comparative insights into CYP1A2, CYP2C9, CYP2C19, or CYP3A4.
[47]	None; general DDIs	FDA Structured Product Labeling (SPL)	Introduced an AG-GCN to predict DDIs by extracting drug interaction data from FDA labels. The model utilizes attention mechanisms within GCNs to identify relevant graph structures in the labels and focus on crucial interaction patterns.	**Key findings:** Achieved improved prediction of DDIs from drug labels through the attention mechanism’s ability to focus on key contextual information. **Notes:** Limited to structured data from FDA labels and does not consider enzyme-specific mechanisms such as CYP-mediated drug metabolism.
[48]	None; general DDIs	DrugBank, TWOSIDES	Proposed a LAGAT model to predict DDIs. LAGAT considers both topological and semantic information from graphs, incorporating link awareness by applying attention mechanisms to the links between nodes (drugs) rather than just the nodes themselves.	**Key findings:** Demonstrated strong predictive accuracy in capturing link-level features, improving DDI prediction significantly. **Notes:** No biochemical insights were provided regarding CYP-mediated interactions, and the model is highly dependent on dataset quality. Scalability remains an issue due to the computational complexity of link-level attention.

### Drug--target interactions

Several graph-based DTI models have been proposed that, while not directly designed for ADMET prediction, have implications for drug metabolism modeling. Ji et al. [49] constructed a multi-molecular network using data from DrugBank and KEGG, employing a random walk algorithm to predict unknown DTIs. The model performed well in addressing the cold-start problem for new targets [49].

Zheng et al. [50] introduced a tripartite heterogeneous network integrating drugs, targets, and biological entities. Their model improved DTI accuracy using Gaussian kernels and regularized least squares [50].

Zhao et al. [51] applied graph representation learning with GNNs to model DTIs on a large scale. The study emphasized molecular and topological features to achieve high predictive performance, particularly in drug repurposing [51].

Li et al. [52] used the LINE network embedding model with a random forest classifier to predict drug--protein interactions, achieving robust results for DTI inference using topological features and collaborative filtering [52]. A comparative summary of these studies is provided in Table 3.

**Table 3:** Summary of graph-based studies on DTIs

Study	CYP isoforms studied	Datasets/Data sources	Methodology	Key findings and Notes
[49]	None directly	DrugBank, KEGG	A novel line network representation method was introduced to predict DTIs. The method constructs a multi-molecular network where drugs, targets, and related entities are nodes, and their interactions are edges. A random walk algorithm explores the graph structure to predict unknown interactions.	**Key findings:** High accuracy (area under the curve (AUC) = 92.33%) in identifying novel DTIs, particularly for targets with few known interactions (cold-start problem). **Notes:** The model’s reliance on existing DTI data limits its generalization to novel compounds, and the lack of a focus on ADMET-specific endpoints, including CYP enzyme interactions, limits its application in pharmacokinetics (PK).
[50]	None directly	DrugBank, KEGG, Universal Protein Resource (UniProt), ChEMBL	Developed a tripartite heterogeneous network for predicting DTIs by integrating three types of nodes---drugs, targets, and biological entities---using Gaussian kernel functions and a regularized least square method to predict interactions.	**Key findings:** High AUC (>0.96) on benchmark datasets for predicting DTIs, especially for challenging cases with sparse known interactions. **Notes:** The model primarily focuses on DTIs and does not consider PK or CYP-mediated metabolism. Its generalization to new chemical entities is limited by existing data.
[51]	None directly	The Binding Database (BindingDB), a publicly accessible resource containing experimentally determined binding affinities for protein--ligand complexes, DrugBank	The study uses graph representation learning to predict DTIs on a large scale. Both drugs and targets are represented as nodes, and interactions are edges in a graph. GNNs extract molecular and topological features from drug-target pairs.	**Key findings:** Achieved high predictive performance (area under the receiver operating characteristic curve (AUROC) = 0.9455, area under the precision--recall curve (AUPR) = 0.9491) in discovering new DTIs, which can be applied for drug repurposing. **Notes:** No enzyme-specific interactions (e.g., CYP) were incorporated, and the model’s reliance on existing datasets limits generalization to novel chemical entities.
[52]	None directly	DrugBank	The study utilized the Large-scale Information Network Embedding (LINE) model and RF algorithm to predict DPIs. The LINE model extracts hidden topological features, and collaborative filtering is used for prediction.	**Key findings:** The LINE-RF combination demonstrated superior performance (AUC = 0.9349, AUPR = 0.9016). **Notes:** Relies on known datasets like DrugBank, and the model struggles with the cold-start problem for novel drugs or proteins. No explicit focus on CYP-mediated interactions.
[53]	None directly	DrugBank, BindingDB, G-Protein Coupled Receptor (GPCR), ChEMBL, KEGG, UniProt	Developed a deep learning model, Independent and Interactive Feature-based Drug--Target Interaction (IIFDTI), to predict drug--target interactions (DTIs) for GPCR & The IIFDTI model combines independent feature extraction (via GAT and CNN) and interactive feature extraction (using a bidirectional encoder--decoder). It captures drug-target relationships and employs attention mechanisms to focus on relevant features.	**Key findings:** While the IIFDTI model is not designed specifically for CYP enzyme prediction, CYP-related targets such as CYP3A4, CYP1A2, and CYP2D6 are identified in the top predictions of the case study on Diacerein (Table 8), with three CYPs validated by literature (PubMed: 18814214). This demonstrates IIFDTI’s capacity to capture biologically meaningful interactions relevant to CYPs. **Notes:** No quantitative performance metrics (e.g., AUC, precision) are reported for CYP-specific endpoints, limiting interpretability for ADMET applications focused on metabolic pathways.
[54]	CYP3A4	DrugBank, KEGG, ChEMBL	Introduced Deep Multi-Modal Path Feature (DeepMPF), a deep learning framework that predicts drug--target interactions (DTIs) by combining multi-modal data (e.g., chemical structure, biological interactions) with meta-path semantic analysis to extract meaningful features.	**Key findings:** DeepMPF demonstrated strong predictive performance for CYP-related DTIs. On the enzyme dataset (including CYPs), it achieved an average AUC of 0.9645 and accuracy of 0.9057 in fivefold cross-validation. Among the top 20 predicted drugs interacting with CYP3A4, 9 were confirmed via DrugBank, showcasing high predictive precision. Molecular docking experiments further validated binding between CYP3A4 and top candidates such as amitriptyline and methadone (e.g., binding energy: $-4.93$ kcal/mol for amitriptyline). **Notes:** While the model is effective for CYP3A4 and other well-studied CYPs, performance may decline for less-studied isoforms or smaller training sets, especially where heterogeneous structural information contributes less.

### Sites of metabolism (SoMs) and adverse drug events

Graph-based models have also been applied to predict metabolic sites and toxicity profiles, both of which are closely tied to ADMET outcomes. Mitchell et al. [55] used GCNs to predict sites of metabolism, outperforming traditional models and enabling structure-based inference for metabolic hotspots [55].

Porokhin et al. [56] developed a GNN model using KEGG data to rank enzymatic products and identify potentially promiscuous SoMs. Their model improved metabolic site prediction, although it was not enzyme-specific [56].

Wu et al. [57] constructed an ADE network model to uncover associations between drugs and adverse effects using real-world data from FAERS, DrugBank, and SIDER. The model successfully identified hidden drug--ADE links using graph-based co-occurrence [57].

While the reviewed models offer valuable insights into DDIs, DTIs, metabolic sites, and toxicity, a common limitation is the lack of integration with enzyme-specific data---particularly those involving CYP isoforms. This omission restricts the applicability of these approaches in ADMET contexts, where metabolic pathways are essential for understanding drug behavior. Bridging interaction prediction models with enzyme-specific features---such as inhibition profiles, substrate recognition, or SoMs---can enable more holistic and clinically relevant ADMET predictions. Incorporating such information, especially within multitask or hybrid learning frameworks, presents an important direction for future research. A summary of these graph-based models and their relevance to ADMET and CYP-mediated processes is provided in Table 4.

**Table 4:** Graph-based models for predicting sites of metabolism and adverse drug events with relevance to ADMET and CYP-mediated processes

Study	CYP isoforms studied	Datasets/Data sources	Methodology	Key findings and Notes
[55]	CYP1A2, 2A6, 2B6, 2C8, 2C9, 2C19, 2D6, 2E1, 3A4	Zaretzki dataset (679 drugs with CYP-specific SOM annotations)	Compared fixed atom fingerprint descriptors (e.g., circular/topological) with learned graph convolutional neural networks (GCNNs) for predicting sites of metabolism (SOMs). Used isozyme-specific and merged datasets with various radii and molecular feature sets.	**Key findings:** GCNNs and fingerprint-based Random Forest models achieved comparable performance. Max MCC: 0.586 (CYP2C9, GCNN), 0.5826 (merged, GCNN), 0.565 (merged, fingerprint). Top-2 accuracy exceeded 85% for most CYPs. CYP3A4 favored small-radius models due to its large binding site. **Notes:** Limited dataset size restricted deep learning potential. SOM annotations varied across sources. GCNNs required more tuning; interpretability was lower than fixed-feature models.
[56]	CYP and non-CYP enzymes; evaluated separately	KEGG RPAIR database (21,016 molecules)	Developed a GNN model (GNN-SOM using Chebyshev convolution) to predict sites of metabolism (SoMs) on atoms and bonds using molecular graphs. Also applied the model to prioritize promiscuous enzymatic products and rank synthetic pathways.	**Key findings:** For CYP-specific reactions, GNN-SOM achieved molecular AUROC of 0.910, atomic AUROC of 0.956, and top-2 correctness of 0.775-significantly outperforming Random Forest and MLP baselines. The model trained on all enzymes performed comparably to CYP-specific models, removing the need for separate predictors. **Notes:** Performance was lower on CYP-mediated reactions compared to non-CYP, partly due to the smaller CYP subset in the dataset (2877 vs. 18,139). Bond-level prediction was also more difficult than atom-level prediction.
[57]	None directly; indirect relevance via metabolism by CYP3A4 for Sonidegib	DrugCentral (FAERS-integrated)	Developed an ADE-ADE network using known drug-ADE associations from DrugCentral (FAERS data) to predict uncharacterized adverse drug events. The model uses a pull-down scoring system to prioritize ADEs based on drug-ADE co-occurrence, with integration of System Organ Class (SOC) classification.	**Key findings:** The model predicted ADEs associated with CYP3A4-metabolized drugs like Sonidegib, including mechanistically plausible effects linked to CYP inhibition. **Notes:** The approach does not include enzyme-specific modeling or CYP interaction data, limiting its direct applicability to CYP-mediated metabolism and toxicities.

## Data sources enriching ADMET prediction models

ADMET prediction models rely on a wide array of datasets that provide information on drug metabolism, pharmacokinetics (PK), toxicity, and drug--drug interactions (DDIs). These datasets---both proprietary and publicly available---serve as the backbone for developing, training, and benchmarking models that aim to improve drug safety and efficacy predictions, particularly for CYP-mediated processes.

**Proprietary datasets**, such as those from Amgen [26], offer high-quality experimental data, including over 250,000 entries related to human liver microsomal (HLM) clearance and cytochrome P450 (CYP) enzyme inhibition (e.g., CYP3A4). These datasets support the development of robust models for predicting metabolic clearance, solubility, bioavailability, and nuclear receptor activity.

**Public repositories** like ChEMBL [58] and DrugBank [59] provide bioactivity data and pharmacological information widely used for predicting metabolic clearance and DDIs. DrugBank versions 4.1.0 to 5.1.1 contain hundreds of thousands of drug--drug and drug--target interactions, including those involving key CYP enzymes such as CYP3A4 and CYP2D6.

**For ADE and toxicity prediction**, the FDA Adverse Event Reporting System (FAERS) [60] and DrugCentral [61] offer real-world, post-marketing surveillance data. FAERS allows models to learn from adverse outcomes, while DrugCentral links adverse events to molecular mechanisms, improving the specificity of ADE prediction models.

**Enzyme-specific datasets** are essential for modeling CYP-mediated drug metabolism. Interaction matrices from DrugBank, KEGG BRITE [62], and BRENDA [63] offer curated information on enzyme--substrate and enzyme--inhibitor relationships across multiple CYP isoforms, enabling accurate prediction of inhibition, activation, and DDIs.

**Structural and chemical bioactivity datasets**, such as ZINC15 [64] and PubChem [65], provide extensive chemical libraries for training models to recognize molecular features impacting ADMET properties. These databases are essential for identifying structure--activity relationships for novel compounds.

**Omics and systems biology data sources**, including UniProt [66] and DisGeNET [67], contribute genetic and proteomic context to ADMET models. They enable incorporation of individual variability, particularly for enzymes like CYP2C9 and CYP2D6, which are known for polymorphic expression impacting drug metabolism.

Table 5 summarizes these data sources, emphasizing their role in enhancing the predictive power and mechanistic understanding of ADMET models.

**Table 5:** Summary of key datasets used in ADMET prediction research

Source	Description	Key characteristics
Amgen Internal	Experimental ADME datasets including CYP3A4 inhibition, HLM clearance, solubility, PXR activity	Proprietary; 250,000+ data points across 13 endpoints
ChEMBL	Public bioactivity data including metabolism and CYP inhibition assays	Broad coverage of small molecules, bioassays, and targets
DrugBank (v4.1–5.1.1)	Drug--target, drug--enzyme, and DDI data	Up to 248,000 interactions; key CYPs like CYP3A4 and CYP2D6
DrugCentral	Curated drug-ADE associations from literature and regulatory sources	90,827 drug-ADE links across 930 drugs and 6,221 ADEs
FAERS	Post-marketing adverse event reports from FDA	Real-world safety data; useful for toxicity and ADE models
ZINC15	Commercially available and drug-like chemical space	Millions of compounds; includes structural and vendor data
PubChem	Bioactivity and structure data for chemicals and drugs	Over 100 million structures; extensive ADMET annotations
BRENDA	Enzyme function, kinetics, and interactions database	CYP isoforms included; supports DDI and metabolism studies
KEGG BRITE	Drug--enzyme interaction mappings and metabolic networks	Includes RPAIR data for site-of-metabolism prediction
Yamanishi Matrix	Drug--target interaction matrices (EN, GPCR, IC, NR)	Compiled from KEGG, DrugBank, SuperTarget, BRENDA
UniProt	Protein sequence and function data	Used for modeling target interactions and genetic variants
DisGeNET	Gene--disease and variant--disease associations	Enables modeling population-level variability in metabolism
GDSC/CTRP	Drug response data in cancer cell lines	Specialized for oncology models; includes synergy predictions

## Conclusion and future directions

Recent advances in graph-based modeling have significantly enhanced ADMET prediction, particularly for major CYP450 isoforms such as CYP3A4, CYP2D6, and CYP2C9. The integration of Graph Neural Networks (GNNs), Graph Convolutional Networks (GCNs), and attention-based mechanisms has not only improved predictive performance, but also contributed to greater interpretability in modeling enzyme--substrate and enzyme--inhibitor interactions. In addition, multi-task learning frameworks, explainable AI (XAI) techniques such as SHAP and integrated gradients, and hybrid models that combine both structural and sequential molecular representations have expanded the methodological landscape for ADMET research.

Despite these advancements, challenges remain. While CYP inhibition prediction has shown robust performance across many models, the prediction of substrates and metabolism-related endpoints continues to be hindered by issues such as data imbalance, inconsistent annotations, and limited experimental validation. Furthermore, interpretability varies widely, particularly when explainability techniques are applied in a post hoc manner without integrating domain-specific knowledge into the model architecture.

Beyond these core modeling tasks, auxiliary graph-based applications targeting drug--drug interactions (DDIs), drug--target interactions (DTIs), sites of metabolism (SoMs), and adverse drug events (ADEs) offer important complementary insights into drug behavior. However, a common shortcoming across many of these studies is the lack of enzyme-specific granularity---especially for CYP isoforms---limiting their applicability for precise metabolic and toxicity predictions.

To move the field forward, several key challenges must be addressed. Many existing models rely on public datasets that suffer from limited coverage of rare CYP isoforms and underrepresentation of structurally diverse compounds. This constraint reduces the models’ generalizability, especially to novel scaffolds or out-of-domain chemical structures. Additionally, although XAI tools can enhance model transparency, their effectiveness is often dependent on how well they are integrated into the model design and the specific architecture employed.

Looking ahead, future research should prioritize expanding and harmonizing datasets to improve chemical diversity and enzyme representation. Integrating proprietary resources, such as Amgen’s HLM data, with public databases like ChEMBL and PubChem will be essential to achieving this goal. In parallel, efforts should focus on developing enzyme-specific models that distinguish between substrates and inhibitors for each isoform, aligning more closely with pharmacological relevance and regulatory needs. Establishing standardized benchmarks and interpretability metrics will also be crucial for fair model evaluation and reproducibility. Furthermore, incorporating experimental validation into model training and refinement workflows will support greater confidence in model predictions and facilitate translational applications. Finally, embracing multimodal learning architectures that integrate omics data, molecular structure, and individual variability---such as genetic polymorphisms---can advance the development of more personalized and accurate predictive tools.

In conclusion, interdisciplinary collaboration among chemists, pharmacologists, data scientists, and clinicians will be essential for operationalizing these models in both clinical and regulatory settings. Graph-based ADMET modeling, when coupled with comprehensive datasets and mechanistic insights, has the potential to revolutionize early-stage drug development and enable safer, more effective, and personalized therapeutic strategies. It is important to note that the findings and insights presented in this review are shaped by the scope of the literature search, language filters, and manual screening parameters applied. Future reviews may benefit from expanding database inclusion to platforms such as Web of Science and IEEE Xplore, incorporating non-English literature, and employing automated pipelines to improve coverage and reduce potential bias.

## Data Availability

No datasets were generated or analysed during the current study.

## Abbreviations

ACC: Accuracy
ADE: Adverse drug event
ADMET: Absorption, distribution, metabolism, excretion, and toxicity
AG-GCN: Attention-gated graph convolutional network
ANN: Artificial neural network
AUC: Area under the curve
AUROC: Area under the receiver operating characteristic curve
AUPR: Area under the precision--recall curve
BA: Balanced accuracy
BindingDB: A web-accessible database of experimentally determined protein--ligand binding affinities
BRENDA: BRaunschweig ENzyme DAtabase
BRITE: Biomolecular relations in information transmission and expression
ChEMBL: Chemical database of bioactive molecules
CMPNN: Communicative message passing neural network---a GNN architecture that enhances message passing by explicitly modeling interactions between nodes and edges.
CTRP: Cancer therapeutics response portal
CYP: Cytochrome P450
DDI: Drug--drug interaction
DeepMPF: Deep multi-modal path feature
DL: Deep learning
DisGeNET: Disease--gene associations database
DPI: Drug--protein interaction
DrugCentral: Online drug information resource created and maintained by division of translational informatics at University of New Mexico.
DTI: Drug--target interaction
ECFP: Extended connectivity fingerprint
FAERS: FDA adverse event reporting system
FDA: Food and Drug Administration
F1: F1-score (harmonic mean of precision and recall)
FP-GNN: Fingerprint-enhanced graph neural network
GCN: Graph convolutional network
GCNN: Graph convolutional neural network
GAT: Graph attention network---a type of GNN that incorporates attention mechanisms into the message-passing process.
GDSC: Genomics of drug sensitivity in cancer
GNN: Graph Neural Network
GPCR: G-protein coupled receptor
GraphNET: A GNN model that incorporates both node and edge features during message passing to improve molecular representation.
GraphSAGE: Graph sample and aggregate---a GNN architecture that learns node embeddings by sampling and aggregating features from local neighborhoods.
HLM: Human liver microsomes
H-ADMET: Hybrid ADMET model using self-supervised learning
IG: Integrated gradients---an XAI method that attributes predictions by integrating gradients from a baseline input to the actual input.
IIFDTI: Independent and interactive feature-based drug--target interaction
KEGG: Kyoto Encyclopedia of Genes and Genomes
LAGAT: Link-aware graph attention network
LINE: Large-scale information network embedding
MACCS: Molecular ACCess System (keys)
MCC: Matthews correlation coefficient
ML: Machine learning
MTL: Multi-task learning
NR: Nuclear receptor
PK: Pharmacokinetics
PubChem: Public Chemical Database from NIH
PXR: Pregnane X receptor
r: Pearson’s correlation coefficient
R^2^: Coefficient of determination
RF: Random forest
RNN: Recurrent neural network
SHAP: Shapley additive explanations
SIDER: Side effect resource maintained by EMBL
SMILES: Simplified molecular input line entry system
Sn: Sensitivity (also known as recall or true positive rate)
SVM: Support vector machine
Tox21: Toxicology in the 21st century (a U.S. government initiative for high-throughput chemical screening)
UniProt: Universal protein resource
XAI: Explainable artificial intelligence
ZINC15: ZINC is not commercial 15
